# Supplementary material for: An Integrated Computational and Experimental Approach to Formulate Tamanu Oil Bigels as Anti-Scarring Agent
Source: Pharmaceuticals (Basel). 2024 Jan 11;17(1):102. doi: 10.3390/ph17010102 (PMC10818744; doi:10.3390/ph17010102)
Supplement: Supplementary file 1 [file pharmaceuticals-17-00102-s001.zip › pharmaceuticals-2704656-supplementary.pdf]

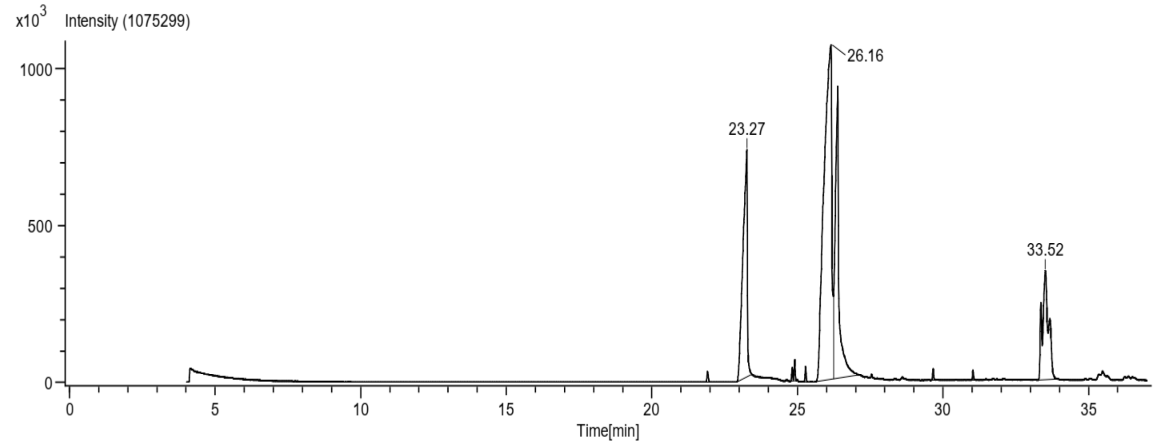

Peak Number	Time [min]	Type	Peak Width(FWH [min]	Area [Intens. * sec]	Height	Description	Start Point		End Point	
							Time[min]	Height	Time[min]	Height
1	21.91	BB	0.0473	101833.53	34459.76		21.85	56	21.99	51
2	23.27	BB	0.1685	7656562.14	724353.37		22.91	336	23.43	25395
3	24.82	BV	0.0404	116360.83	45187.20		24.77	2589	24.87	3629
4	24.91	VB	0.0400	173666.77	68956.47		24.87	3629	24.96	4600
5	25.28	BB	0.0405	129556.19	51005.85		25.23	1019	25.35	760
6	26.16	BV	0.3166	19826989.67	1065966.20		25.63	1464	26.24	10628
7	26.38	VB	0.1161	8384325.97	931228.59		26.24	10628	27.10	23477
8	29.66	BB	0.0381	92273.96	37011.47		29.60	6023	29.74	7223
9	31.03	BB	0.0342	77637.72	33086.16		30.97	6995	31.13	7155
10	33.52	BB	0.1440	5238944.12	348448.68		33.27	7281	33.95	10301

Figure S1: Representative GC chromatogram of tamanu oil components

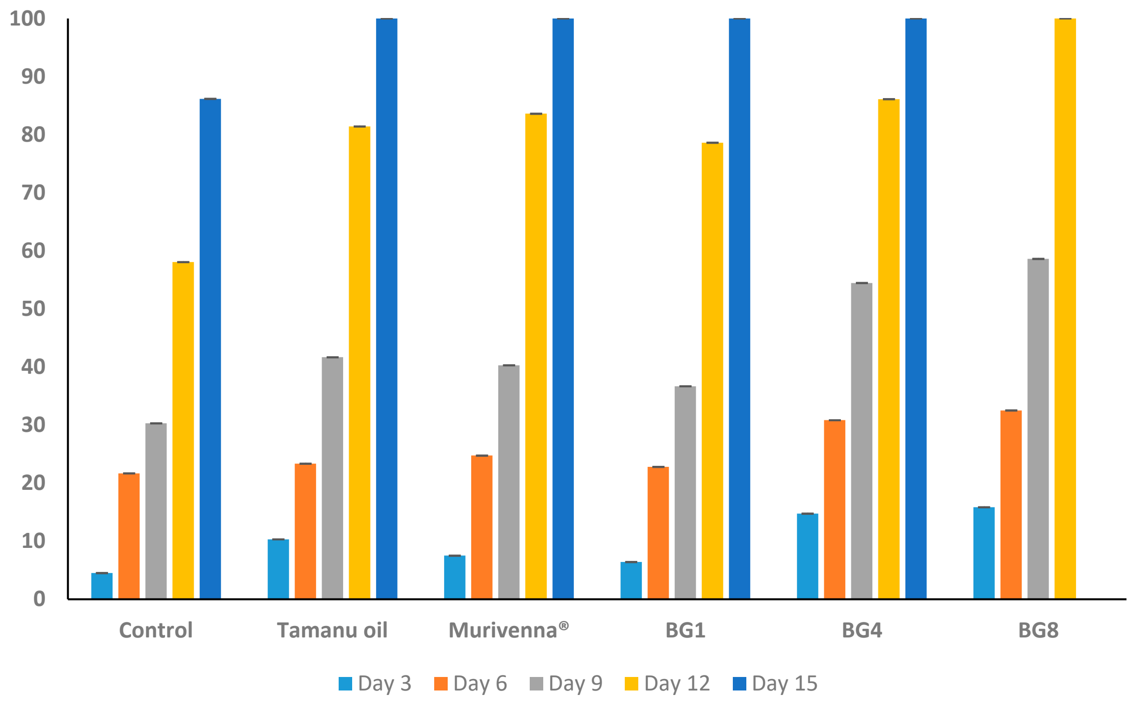

Figure S2: Graphical representation of % Wound Contraction

Groups	(H& E)	Masson's trichome (MT)
**Control**	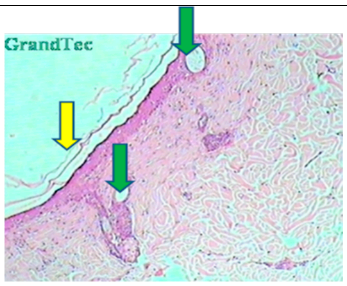	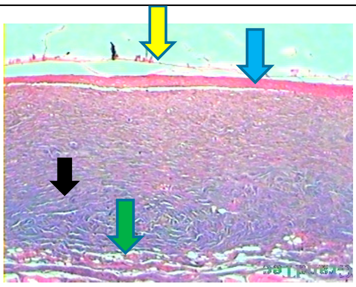
**Tamanu oil**	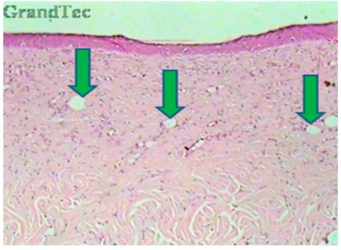	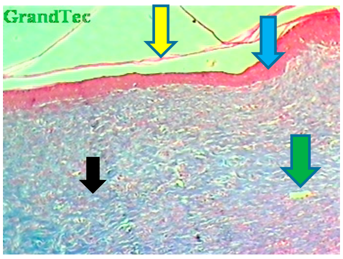
**Murivenna®**	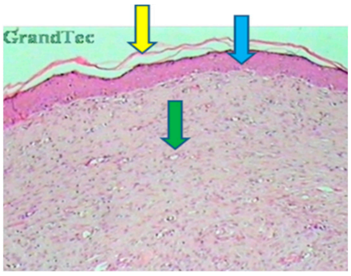	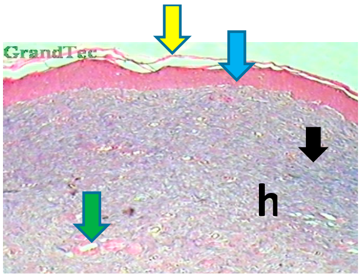
**BG1**	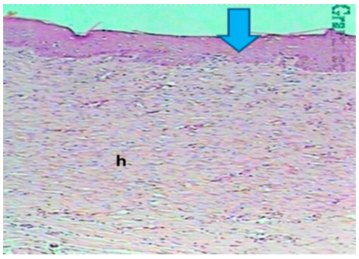	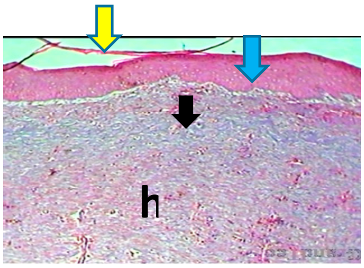
**BG4**	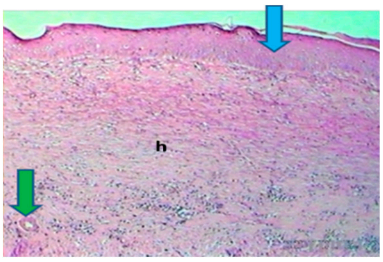	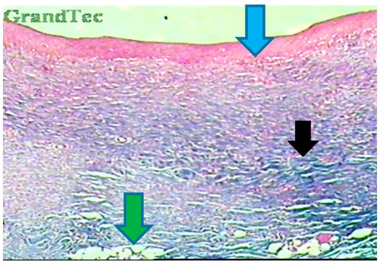

**BG8**	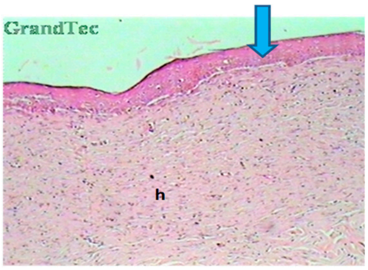	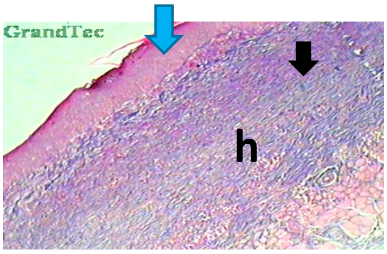

Figure S3: Photomicrographs (100x) showing H&E stain and MT stain section of skin tissues at day 15 for control, standard and formulation treated groups

(**Yellow**- re-epithelialization, **Green**- irregular connective tissue, **Blue**- epidermis, **Black**- collagen formation and **h**- healed area with multiple layers of fibrous connective tissue)

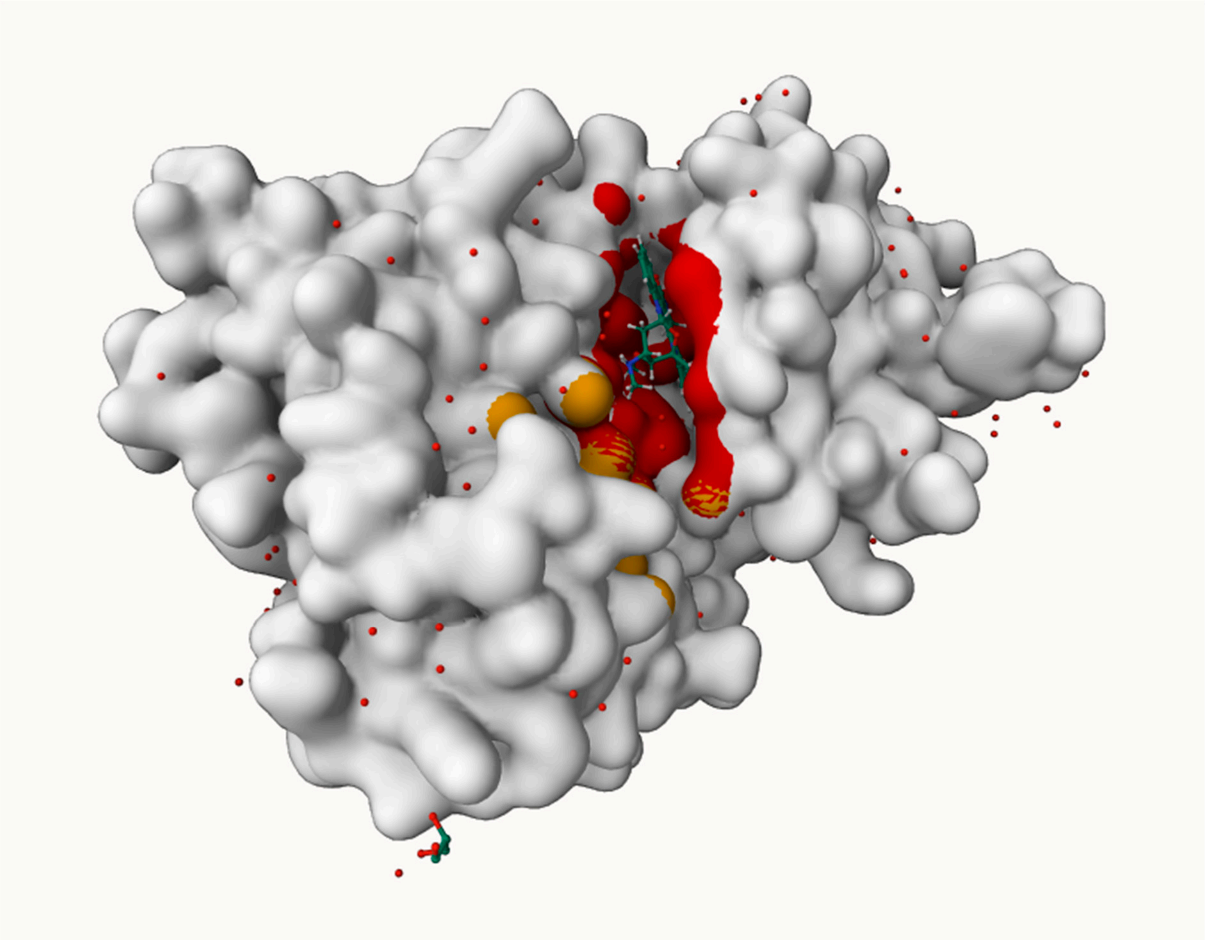

Figure S4: TGF-β type 1 kinase (5E8W) with binding site

Table S1: Molecular docking results (Kcal/mol)

Compounds	Structure	5KF4	3UTZ	3V96	5K5X	6T9D	4D2R	7SZL	2L3Y
**Calophyllolide**	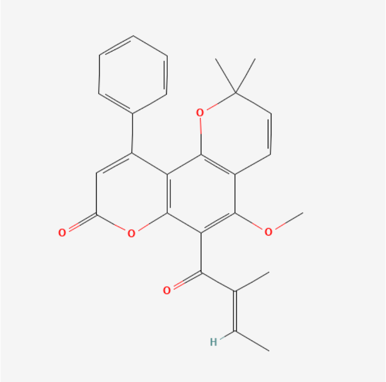	-4.9	-4.5	-4	-4.4	-4.7	-4.6	-4.8	-4.9
**Inophyllum C**	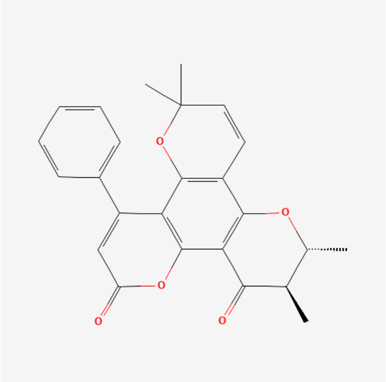	-5.8	-3.1	-7	-7.6	-5.4	-7.5	-7.2	-5.6

**Calanolide A**	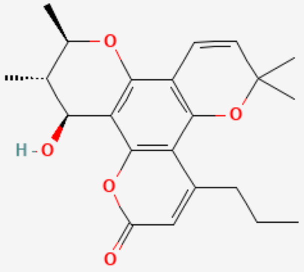	-4	-6.7	-4.4	-6.5	-6	-7.2	-7.7	-6.1
**Oleic acid**	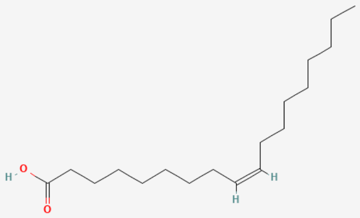	-4.5	-5.9	-4	-5.2	-6.4	-6.5	-6.8	-5.6
**Linoleic acid**	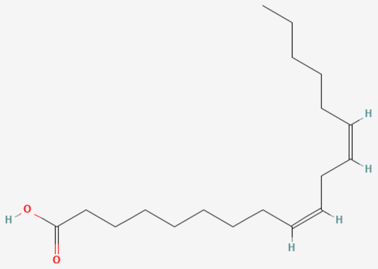	-5.8	-5.8	-6.2	-7	-7.6	-7.6	-5.8	-7.6

**Palmitic acid**	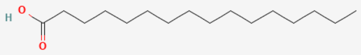	-5.8	-4.6	-5.3	-6.6	-7.2	-7.2	-6.1	-6.7
**4-Norlanosta-17(20),24-diene-11,16-diol-21-oic acid, 3-oxo-16,21-lactone**	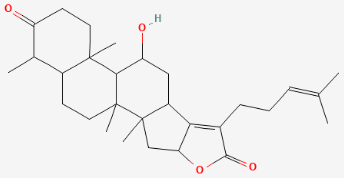	-4.9	-6	-4.7	-5.8	-4.8	-6.4	-6.1	-6.9
**Hyenic acid**	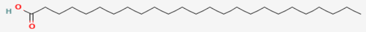	-5.5	-4.9	-4.4	-4.8	-5.1	-4.9	-5	-5.9

Table S2: Molecular properties of Ligands (constituents of Tamanu oil)

**Properties/ Drugs**	**Molecular Weight**	**LogP**	**No. of Rotatable Bonds**	**No. of H-Bond Acceptors**	**No. of H-Bond Donors**	**Surface Area (m^2)**
**Calophyllolide**	416.473	5.8017	4	5	0	180.154
**Inophyllum C**	402.446	5.2439	1	5	0	173.473
**Calanolide A**	370.445	4.3801	2	5	1	158.144
**4-Norlanosta-17(20),24-diene-11,16-diol-21-oic acid, 3-oxo-16,21-lactone**	454.651	5.7834	3	4	1	198.78

Table S3: Predicted ADMET properties of compounds

**Properties**	**Calophyllolide**	**Inophyllum C**	**Calanolide A**	**4-Norlanosta-17(20),24-diene-11,16-diol-21-oic acid, 3-oxo-16,21-lactone**
**Water solubility (log mol/L)**	-5.699	-4.952	-4.982	-5.716
**Caco2 permeability (log Papp in 10-6 cm/s)**	1.121	1.119	0.757	0.605
**Skin Permeability (log Kp)**	-2.702	-2.729	-3.013	-3.253
**P-glycoprotein substrate**	No	No	Yes	No
**P-glycoprotein I inhibitor**	Yes	Yes	Yes	Yes
**P-glycoprotein II inhibitor**	Yes	Yes	Yes	Yes
**VDss (human)(log L/kg)**	0.47	0.169	0.371	-0.124
**Fraction unbound (human)(Fu)**	0.147	0.165	0.103	0
**BBB permeability (log BB)**	-0.823	-0.453	-0.322	-0.56
**CNS permeability (log PS)**	-1.678	-1.606	-1.82	-1.59
**CYP2D6 substrate**	No	No	No	No
**CYP3A4 substrate**	Yes	Yes	Yes	Yes
**CYP1A2 inhibitor**	Yes	Yes	Yes	No
**CYP2C19 inhibitor**	Yes	Yes	Yes	No
**CYP2C9 inhibitor**	Yes	Yes	Yes	No

**CYP2D6 inhibitor**	No	No	No	No
**CYP3A4 inhibitor**	Yes	Yes	Yes	No
**Total Clearance(log ml/min/kg)**	0.757	0.589	0.506	0.331
**Renal OCT2 substrate**	No	No	No	No
**AMES toxicity**	No	No	No	No
**Max. tolerated dose (human)(log mg/kg/day)**	0.255	0.11	-0.315	-0.535
**hERG I inhibitor**	No	No	No	No
**hERG II inhibitor**	Yes	Yes	Yes	No
**Hepatotoxicity**	No	Yes	No	No
**Skin Sensitisation**	No	No	No	No

Table S4: Period of epithelialization in all groups

Sl. no.	Groups	Period of Epithelialization (days)
1.	Control	12.83±0.30
2.	Tamanu oil	10.16 ±0.47**
3.	Murivenna®	10.00±0.36**
4.	BG1	11.50±0.42*
5.	BG4	8.16±0.60**
6.	BG8	6.66±0.66**

Data are expressed as Mean ± SEM (n=6) and analysed by one-way ANOVA method of variance followed by Dunnett test for multiple comparison. ns p>0.05, * p<0.05, ** p<0.01, in comparison to control. (Where, ns= non-significant, * moderately significant,

Table S5: Formulation of Bigels

	BG1	BG2	BG3	BG4	BG5	BG6	BG7	BG8
**OLEOGEL PHASE**								
**Tween 20 (mL)**	3	3	3	3	3	3	3	3
**Tamanu oil (mL)**	5	10	15	20	5	10	15	20
**HYDROGEL PHASE**								
**Micronized Xanthan gum (g)**	1	1	1	1	2	2	2	2
**Geogard® ECT (mL)**	1	1	1	1	1	1	1	1
**Vanilla fragrance oil (mL)**	0.5	0.5	0.5	0.5	0.5	0.5	0.5	0.5
**Water (mL)**	q.s 100mL							
